# Effect of Hypoxia on Gene Expression in Cell Populations Involved in Wound Healing

**DOI:** 10.1155/2019/2626374

**Published:** 2019-08-22

**Authors:** Sarah D'Alessandro, Andrea Magnavacca, Federica Perego, Marco Fumagalli, Enrico Sangiovanni, Mauro Prato, Mario Dell'Agli, Nicoletta Basilico

**Affiliations:** ^1^Dipartimento di Scienze Biomediche, Chirurgiche e Odontoiatriche, Università degli Studi di Milano, Milan, Italy; ^2^Dipartimento di Scienze Farmacologiche e Biomolecolari, Università degli Studi di Milano, Milan, Italy; ^3^Dipartimento di Scienze della Sanità Pubblica e Pediatriche, Università degli Studi di Torino, Turin, Italy; ^4^Dipartimento di Neuroscienze, Università degli Studi di Torino, Turin, Italy

## Abstract

Wound healing is a complex process regulated by multiple signals and consisting of several phases known as haemostasis, inflammation, proliferation, and remodelling. Keratinocytes, endothelial cells, macrophages, and fibroblasts are the major cell populations involved in wound healing process. Hypoxia plays a critical role in this process since cells sense and respond to hypoxic conditions by changing gene expression. This study assessed the in vitro expression of 77 genes involved in angiogenesis, metabolism, cell growth, proliferation and apoptosis in human keratinocytes (HaCaT), microvascular endothelial cells (HMEC-1), differentiated macrophages (THP-1), and dermal fibroblasts (HDF). Results indicated that the gene expression profiles induced by hypoxia were cell-type specific. In HMEC-1 and differentiated THP-1, most of the genes modulated by hypoxia encode proteins involved in angiogenesis or belonging to cytokines and growth factors. In HaCaT and HDF, hypoxia mainly affected the expression of genes encoding proteins involved in cell metabolism. This work can help to enlarge the current knowledge about the mechanisms through which a hypoxic environment influences wound healing processes at the molecular level.

## 1. Introduction

Wound healing is a complex multistep and multicellular biological process, traditionally divided into four overlapping phases known as haemostasis, inflammation, proliferation, and remodelling [1]. Inflammation and hypoxia are mutually interdependent: hypoxia-elicited inflammation is implicated in the outcomes of a wide range of human diseases. The delay in wound healing and wound chronicity are directly linked to persistent inflammation. On the other hand, inflammatory states are frequently characterised by tissue hypoxia, or by the stabilisation of hypoxia-dependent transcription factors [2, 3]. The healing process is regulated by multiple signals such as growth factors, cytokines, chemokines, matrix metalloproteinases (MMPs) and extracellular macromolecules [4, 5]. Upon skin injury, innate immune cells (neutrophils and macrophages) are recruited to the site of injury to remove cellular debris and to secrete mediators able to activate keratinocytes, endothelial cells and fibroblasts. Angiogenesis is crucial to ensure an adequate supply of blood for tissue repair and wound healing [6]. Endothelial cells proliferate, demolish basement membrane and migrate to form new blood vessels starting from the ones located at wound edges. Fibroblasts produce collagen, elastin, proteoglycans and other glycoproteins of the extracellular matrix, which then mature outside the cells. Some fibroblasts develop into myofibroblasts that cause contraction of the wound. Keratinocytes proliferate and migrate from the edges of the wound to restore a confluent epithelium. Migration and proliferation of all the cell types is regulated by complex mechanisms of inhibition and stimulation by growth factors and chemoattractants.

Keratinocytes, endothelial cells, macrophages and fibroblasts are indeed the major cell populations involved in wound healing processes and all of these cells cross-talk with one another to restore normal tissue [7]. Oxygen is a key regulator of ordered wound healing since it is required for epithelialisation, angiogenesis, collagen deposition, and resistance to infection [8]. Hypoxia in wound is mainly caused by the disruption of blood vasculature causing impairment of oxygen delivery to the site of injury. Moreover, the rapid recruitment of inflammatory cells increases oxygen demand to achieve phagocytosis and microbial killing. Reduced oxygen supply leads to chronic hypoxia along with inadequate healing or chronic wounds. Cells sense hypoxia and can alter gene expression changing their metabolism in order to promote cell survival. The transcriptional response is mainly mediated by hypoxia-inducible factor 1 (HIF-1) which regulates the transcription of hundreds of genes that promote cell survival in hypoxia. Different genes involved in regulation of metabolism, cell proliferation and angiogenesis are modulated by hypoxia, but gene expression profiles in response to hypoxia differ among different cell populations.

This study aimed at assessing the gene expression responses to hypoxia in four different cell types involved in wound healing. In particular, cell processes/functions relevant for wound healing, namely angiogenesis, metabolism, cell growth and proliferation, apoptosis, transcription and signalling, were identified. The expression of 77 genes involved in these processes were explored in vitro, using cell models of keratinocytes, endothelial cells, macrophages, and fibroblasts.

This study, addressing the cell-specific responses to hypoxia, may help to better understand the regulation of gene expression profile in different cell populations, and it may provide insight on the role of hypoxia in wound healing.

## 2. Materials and Methods

### 2.1. Reagents

All reagents were from Sigma Aldrich S.r.l. (Milano, Italy), unless otherwise stated. All reagents for cell culture were from EuroClone S.p.A (Pero, Italy), unless otherwise stated. All reagents for RT-qPCR were from QIAGEN S.r.l. (Milano, Italy), unless otherwise stated.

### 2.2. Cells Cultures

HMEC-1, a long-term cell line of dermal microvascular endothelial cells (HMEC-1) immortalised by SV 40 large T antigen [9], was maintained in MCDB-131 medium (Invitrogen, Carlsbad, CA) supplemented with 10% heat-inactivated foetal calf serum (FCS) (HyClone, South Logan, UT), 10 ng/ml of epidermal growth factor (PeproTech, Rocky Hill, NJ), 1 *μ*g/ml of hydrocortisone, 2 mM glutamine, 100 units/ml of penicillin, 100 *μ*g/ml of streptomycin, and 20 mM HEPES buffer, pH7.4.

THP-1 cells (human acute monocytic leukaemia cell line) were maintained in RPMI supplemented with 10% heat-inactivated FCS, 50 *μ*M 2-mercaptoethanol, 10 *μ*M Sodium Pyruvate, 20 mM HEPES, and 2 mM glutamine. In order to achieve differentiation into macrophages, cells were treated with 10 ng/ml phorbol myristate acetate (PMA) for 72 h.

HaCaT (CVCL-0038, Cell Line Service GmbH, Germany), a spontaneously transformed immortal keratinocyte cell line from adult human skin, were maintained in DMEM supplemented with 10% heat-inactivated FCS, 100 U/ml penicillin-streptomycin (GibcoTM, Life Technologies Italia, Monza, Italy), 2 mM L-glutamine (Life Technologies Italia, Monza, Italy).

HDF, normal adult human primary dermal fibroblasts, were maintained in DMEM supplemented with 10% heat-inactivated FCS, 100 U/ml penicillin-streptomycin (Life Technologies, Italy), and 2 mM glutamine (Life Technologies, Italy).

All the cell lines were cultured in standard conditions, at 37°C in a humidified atmosphere containing 5% CO_2_.

### 2.3. Cell Treatment

HMEC-1 were seeded at 2*∗*10^5^ cells/well in 6-well flat bottom tissue culture clusters and incubated for 72 hours to obtain adhesion to the plastic. THP-1 were seeded at 5*∗*10^5^ cells/well in 24-well flat bottom tissue culture clusters and incubated with PMA (10 ng/ml) for 72 hours to achieve differentiation into macrophages. HaCaT were seeded at 6*∗*10^4^ cells/well in 24-well flat bottom tissue culture clusters and incubated for 72 hours. HDF were seeded at 12*∗*10^4^ cells/well in 24-well flat bottom tissue culture clusters and incubated for 72 hours.

Cells were then incubated for 24 hours in hypoxic or normoxic condition. A Hypoxia Incubator Chamber (StemCells Technologies) was filled with a gas mixture consisting of 1% O_2_, 5% CO_2_, 94% N_2_ for 5 minutes at a rate of 10 L/min to achieve hypoxia, according to an established protocol which was previously shown to induce HIF-1 activation in cells [10]. At the end of incubation, mRNA from cell cultures was isolated.

### 2.4. RNA Extraction

Samples (10^∧^6 cells) were lysed in QIAzol lysis reagent. Total RNA was extracted from cell lysates using the miRNeasy Mini Kit following the manufacturer's protocol. A set of RNase free DNase was used to provide efficient on-column digestion of genomic DNA. Total RNAs were eluted in 35 *μ*L nuclease-free water. The RNA concentration and quality were assessed using the NanoDrop ND-1000 spectrophotometer (Thermo Fisher Scientific Italia, Rodano, Italy). Sample purity was estimated by measuring A260/280 and A260/230 ratios of spectrophotometric absorbance to check for possible copurified contaminants during the RNA isolation.

### 2.5. RT-qPCR

cDNA synthesis and genomic DNA elimination were performed with the RT^2^ First Strand Kit, using 400 ng of total RNA for each sample. Samples were quantified using Custom RT2 Profiler PCR Arrays (QIAGEN) by real-time PCR using a CFX384 real-time system instrument (Bio-Rad Laboratories S.r.l., Segrate, Italy) with the RT^2^ SYBR Green qPCR Mastermix. The manufacturer's instructions were strictly followed.

Expression data were normalised to the housekeeping gene RPLP0 (Ribosomal Protein Lateral Stalk Subunit P0). The relative quantification in gene expression was determined using a modified ΔΔCt method based on Pfaffl equation [11].

### 2.6. Cytotoxicity and Viability Assays

HMEC-1 was seeded at 10^4^ cells/well in 96-well flat bottom tissue culture clusters and incubated overnight to obtain adhesion to the plastic. THP-1 were seeded at 10^5^ cells/well in 96-well flat bottom tissue culture clusters and incubated with PMA (10 ng/ml) for 72 hours to achieve differentiation into macrophages. HaCaT were seeded at 6*∗*10^4^ cells/well in 24-well flat bottom tissue culture clusters and incubated for 72 hours. HDF were seeded at 12*∗*10^4^ cells/well in 24-well flat bottom tissue culture clusters and incubated for 72 hours. Cells were then incubated for 24 hours in hypoxic or normoxic condition as described above.

### 2.7. MTT Assay

Cell viability was evaluated using the 3-(4,5-dimethylthiazol-2-yl)-2,5-diphenyltetrazolium bromide (MTT) assay. After incubation of cells in normoxic or hypoxic conditions, 10% V/V of a 5 mg/mL solution of MTT in PBS were added to the cells for 3 hours at 37°C in the dark. The supernatants were then discarded, and the dark blue formazan crystals dissolved using 100 *μ*L of lysis buffer containing 20% (w/V) sodium dodecylsulfate, 40% N, N-dimethylformamide (pH 4.7 in 80% acetic acid). The plates were then read on a Synergy 4 (Biotek®) microplate reader at a test wavelength of 550 nm and at a reference wavelength of 650 nm.

### 2.8. LDH Assay

The potential cytotoxic effect of hypoxia was measured as the release of lactate dehydrogenase (LDH) from cells into the extracellular medium using the LDH-Cytotoxicity Colorimetric Assay Kit II (BioVision, Segrate, Italia) following the manufacturer's instructions. LDH was measured both in the extracellular medium and inside the cells. To measure the extracellular LDH, 10 *μ*L of supernatants were mixed with 100 *μ*L of LDH reaction mix. To measure intracellular LDH, 10 *μ*L of Cell Lysis Solution were added to the wells and 11 *μ*L of the lysate were mixed with 100 *μ*L of LDH reaction mix. Samples were incubated for 30 minutes at room temperature in the dark. Absorbance was then read at 450 nm with a reference wavelength of 650 nm using Synergy 4 (Biotek®) microplate reader. Percent cytotoxicity was calculated following the equation modified by Smith [12].

### 2.9. Statistical Analysis

All data were obtained from at least three independent experiments. Results are shown as means ± standard deviation. Differences between normoxia and hypoxia were analysed by two-tailed Student's t-test.

## 3. Results and Discussion

### 3.1. Effect of Hypoxia on Cell Viability

**Figure 1:**
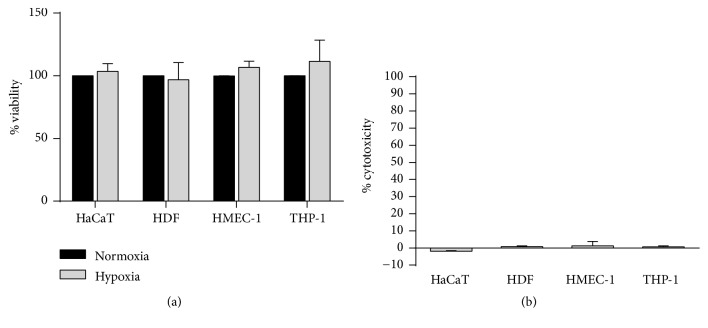
Hypoxia effect on cell viability. HaCaT, HDF, HMEC-1 and differentiated THP-1 were incubated for 24 h in normoxia (black bars) or hypoxia (1% O2, gray bars). The percentage of cell viability was measured using the MTT assay (panel (a)), whereas the percentage of cytotoxicity was calculated measuring the release of LDH (panel (b)). Data are shown as mean ± standard deviation of three independent experiments. Two-tailed Student's t-test was performed to compare viability and cytotoxicity in normoxia vs hypoxia.

Cells were incubated in hypoxic condition for 24 hours and viability was measured by MTT and LDH assays. A direct toxicity was not observed since cytotoxicity, measured as the release of LDH from cell monolayers, or viability, measured as MTT metabolism, were comparable in hypoxic and normoxic conditions in all the cell types (Figure 1). Cell morphology was not macroscopically altered by hypoxia.

### 3.2. Effect of Hypoxia on Gene Expression

In response to hypoxia, cells undergo a metabolic reprogramming orchestrated by hypoxia-inducible factors (HIFs) which translocate to the nucleus and activate the transcription of hundreds of target genes. Nevertheless, the transcriptional program in response to a hypoxic challenge extensively varies among different cell types [13, 14].

In this work, the gene expression program in response to hypoxia in cell types involved in wound healing was characterised. The expression of 77 genes was measured by RT-qPCR after 24 h of cell exposure to hypoxia. The transcript levels for each gene in cells cultured under hypoxia (1% O_2_) were compared to the transcript levels in the same cell type cultured in normoxic conditions (~21% O_2_). Seven common genes induced by hypoxia in all the tested cells were identified. These common upregulated genes encode proteins involved in glycolytic metabolism (TPI1, Triosephosphate isomerase 1; HK2, hexokinase 2), cell proliferation and apoptosis (BNIP3, BCL2 Interacting Protein 3), angiogenesis (VEGFA, Vascular endothelial growth factor A; ANGPTL4, Angiopoietin like 4), or transcription and signalling (ANKRD37, Ankyrin Repeat Domain 37; BHLHE4, Basic Helix-Loop-Helix family member e40). Among the selected genes, only few were downregulated and none was downregulated in all the four cell lines.

The expression of 14 genes was not significantly modulated by hypoxia in any tested cell types. These genes encode chemokines and cytokines (*CCL11*, C-C Motif Chemokine Ligand 11;* CXCL1*, C-X-C Motif Chemokine Ligand 1;* CXCL10*, C-X-C Motif Chemokine Ligand 10;* CXCL5*, C-X-C Motif Chemokine Ligand 5), growth factors and receptors (*EGF*, Epidermal Growth factor;* FGF1*, Fibroblast Growth Factor 1;* IGF1*, Insulin Like Growth Factor 1;* ERBB2*, Erb-B2 Receptor Tyrosine Kinase 2;* S1PR1*, Sphingosine-1-phosphate receptor 1), transcription and signalling factors (*HNF4A*, Hepatocyte Nuclear Factor 4 Alpha;* ID1*, Inhibitor of DNA binding 1,* HLH* protein), as well as proteins involved in angiogenesis (*COL18A1*, Collagen type XVIII alpha 1 chains;* LECT1*, chondromodulin) and coagulation (*THBS2*, Thrombospondin 2).

Two genes* CXCL9* (C-X-C Motif Chemokine Ligand 9) and* IFNG* (Interferon Gamma) were not expressed in either cell type.

**Figure 2:**
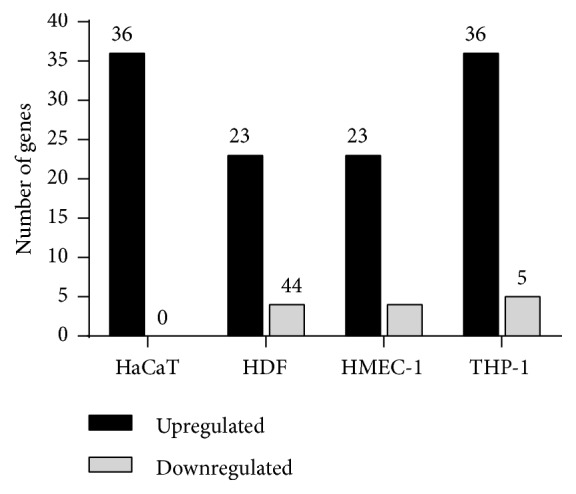
Regulation of gene expression. HaCaT, HDF, HMEC-1 and THP-1 were incubated for 24 h in normoxia or hypoxia. Histograms represent the number of genes significantly up-regulated (black bars) or downregulated (grey bars) in each cell type.

Depending on the cell types, a different number of genes were up- or downregulated. The detailed number of hypoxia-regulated genes in each cell types are shown in Figure 2. Raw data are presented in Supplementary [Supplementary-material supplementary-material-1].

Then we focus on specific sets of hypoxia-regulated genes, depending on the function of the encoded protein. Groups were labeled “angiogenesis”, “apoptosis/cell cycle”, “cytokines/chemokines”, “growth factors/receptors”, “coagulation”, “transcription/signaling factors”, “glycolytic metabolism”, “non-glycolytic metabolism”. However, the wound healing process involves complex interactions between angiogenesis, inflammation, coagulation and extracellular matrix deposition; therefore some genes included in a given set may be also relevant in others processes.

### 3.3. Angiogenesis

**Figure 3:**
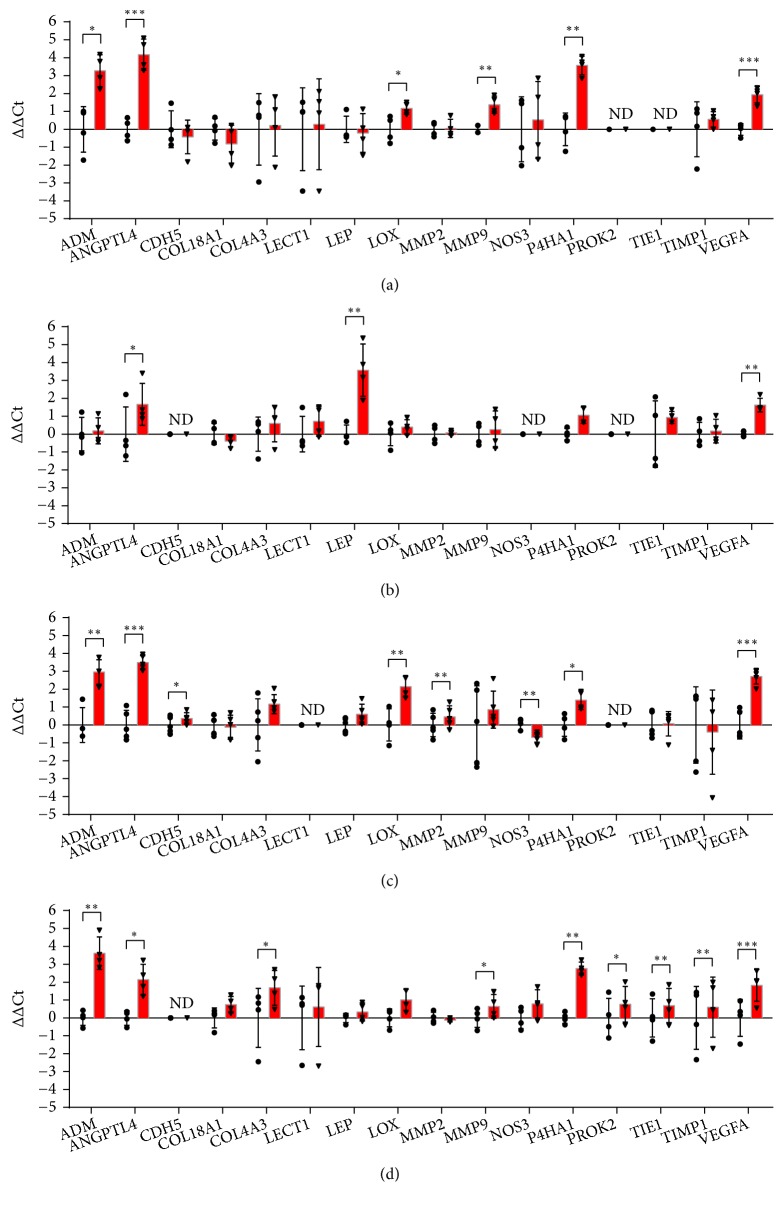
RT-qPCR analysis of genes involved in angiogenesis after 24 hours of incubation in normoxia or hypoxia in HaCaT (a), HDF (b), HMEC-1 (c) and THP-1 (d). The results are expressed as ∆∆Ct after normalization on RPLP0 housekeeping gene. Data are shown as mean ± standard deviation and as single values distribution of four independent experiments. Circles (●) and triangles (▼) represent ∆∆Ct values in normoxia and hypoxia, respectively. Statistical analysis was performed using the two-tailed Student's t-test comparing, for each gene, the expression in hypoxia versus normoxia (*∗*p-value < 0,05; *∗∗* p-value < 0,01; *∗∗∗*p-value < 0,001).

The expression of 16 genes coding proteins strictly involved in angiogenesis was analysed (Figure 3). Two genes (MMP2, Matrix Metallopeptidase 2 and CDH5, VE-cadherin) were significantly up-regulated by hypoxia only in HMEC-1, whereas COL4A3 and LEP were specifically increased in THP-1 and HDF, respectively.

*VEGF-A* is produced by many cell types involved in wound healing, and it plays a key role not only in angiogenesis, but also in epithelisation and collagen deposition [15]. VEGF-A induces endothelial cell proliferation and migration stimulating chemotaxis and vasodilatation. VEGFA gene expression is increased by hypoxia in different cell types [15] and, as expected, it was upregulated in all the cell lines used in this work.* ANGPTL4*, the other gene up-regulated in all the cells tested, is induced by HIF-1*α* in hypoxic conditions [16].* ANGPTL4* encodes Angiopoietin Like 4, a secreted factor belonging to a superfamily of proteins implicated in the regulation of metabolism, inflammation and angiogenesis [17]. Angiopoietin Like 4 improves angiogenesis by disrupting the integrity of vascular junctions and by inducing vascular leakage and plays an important role in wound repair [18, 19]. Our data indicate that under hypoxia all the cells implicated in wound healing participate in the induction of both VEGFA and ANGPTL4 to accelerate wound repair (Figure 3).

Most of the analysed genes are involved in extracellular matrix deposition and remodelling. The balance between matrix metalloproteinases (MMPs) and their inhibitors (tissue inhibitors of metalloproteinases, TIMPs) is a key process in wound healing. Hypoxia increased the expression of* MMP2* in HMEC-1 (Figure 3(c)) and of* MMP9* in HaCaT and THP-1 (Figures 3(a) and 3(d)). The enhanced expression of MMP2 is consistent with previous studies describing the induction of MMP-2 protein levels and activity in HMEC-1 by hypoxia [20], but in contrast with the downregulation observed by Loboda and colleagues using macroarray analysis [21]. The modulation of MMP-9 in keratinocytes cultured in hypoxia is controversial: MMP-9 is increased by hypoxia in human keratinocytes [22] but decreased in HaCaT [23]. Xia and colleagues have proposed that hypoxia-dependent regulation of MMP production varies depending on the donor's age [24]. Interestingly, both* MMP9* and* TIMP1* were upregulated in differentiated THP-1, indicating that hypoxia induces a coordinated mechanism able to activate matrix degradation and to prevent excessive proteolysis at the same time.* COL18A1* and* COL4A3* encode chains of XVIII and IV collagen types. Hypoxia did not modulate their expression except for COL4A3, which was significantly up-regulated in THP-1 (Figure 3(d)). Although macrophages are mostly involved in matrix degradation, their ability to express all collagen mRNA was described [25]. The cross-linking of collagens is catalysed by lysyl oxidases [26, 27], extracellular copper enzymes. Lysyl oxidase (*LOX*) is a hypoxia-responsive factor associated with the malignant progression of carcinoma [28]. In our work, hypoxia induced an increase of the* LOX* gene expression in HMEC-1 and HaCaT, although in the latter cell line the expression level was low (Figures 3(a) and 3(c)). Increased expression of* P4HA1*, encoding one of the isoforms of collagen prolyl 4-hydroxylases (P4Hs), was observed in all cell types except for HDF (Figure 3). This enzyme is involved in the biogenesis of collagen into stable, mature, triple helical form [29]. Previous studies have shown that the expression of* P4HA1* mRNAs is increased under hypoxic conditions in various cell types [30, 31]. Altogether, these data confirm and expand knowledge on the role of hypoxia in matrix remodeling during wound healing process [32].

ADM (adrenomedullin) is an autocrine and paracrine vasoactive peptide with hypotensive and immune-modulating activity [33] able to promote angiogenesis by inducing proliferation and migration of endothelial cells [34].* ADM *gene is a HIF-responsive gene [35] in a variety of cell lines, including HMEC-1 [36]. Here,* ADM* expression was enhanced in all cell types except for HDF (Figure 3).

Low level expression of* LEP* (leptin) gene was observed in all the cell types except HDF, where the expression was increased by hypoxia (Figure 3). The* LEP* gene encodes a protein that is secreted by white adipocytes into the circulation and plays a major role in the regulation of energy homeostasis. This protein also has endocrine functions and is involved in the regulation of immune and inflammatory responses, haematopoiesis, angiogenesis, reproduction, bone formation and wound healing [37, 38].

*CDH5* and* NOS3* genes are specifically expressed in endothelium.* CDH5* encodes VE-cadherin, one of the most important cell junction proteins involved in vessel organization [39]. VE-cadherin is also expressed in tumours, where it is induced by hypoxia [40]. In our endothelial model* CDH5* is significantly increased by hypoxia, consistently with previous data [21].* NOS3* encodes endothelial nitric-oxide synthase (*eNOS*), an enzyme constitutively expressed in endothelial cells. Among those analysed in this work, it is one of the few genes significantly downregulated in HMEC-1 upon 24 h hypoxia (Figure 3(c)). Also the orphan receptor* TIE1* (tyrosine kinase with immunoglobulin like and EGF like domains 1) is specific of endothelial cells. It is involved in angiogenesis since it inhibits angiopoietin 1 signaling interacting with the endothelial receptor tyrosine kinase Tie2.* TIE1* was significantly increased by hypoxia only in THP-1 (Figure 3(d)).

*PROK2* encodes prokineticin 2, which is increased in wound healing as demonstrated in human skin biopsies [41]. However, in our model* PROK2* was expressed only in differentiated THP-1 where it was increased by hypoxia (Figure 3(d)). The lack of* PROK2* expression in the other cell types may indicate that the induction of this gene requires other stimuli such as cell-cell interactions.

*LECT1* is not relevant to the skin model, since it encodes Chondromodulin, which promotes chondrocyte growth and inhibits angiogenesis in cartilage [42].* LECT1* was not expressed in HMEC-1. In the other three cell types,* LECT1* was expressed at low level and not modulated by hypoxia (Figure 3).

### 3.4. Apoptosis and Cell Cycle

**Figure 4:**
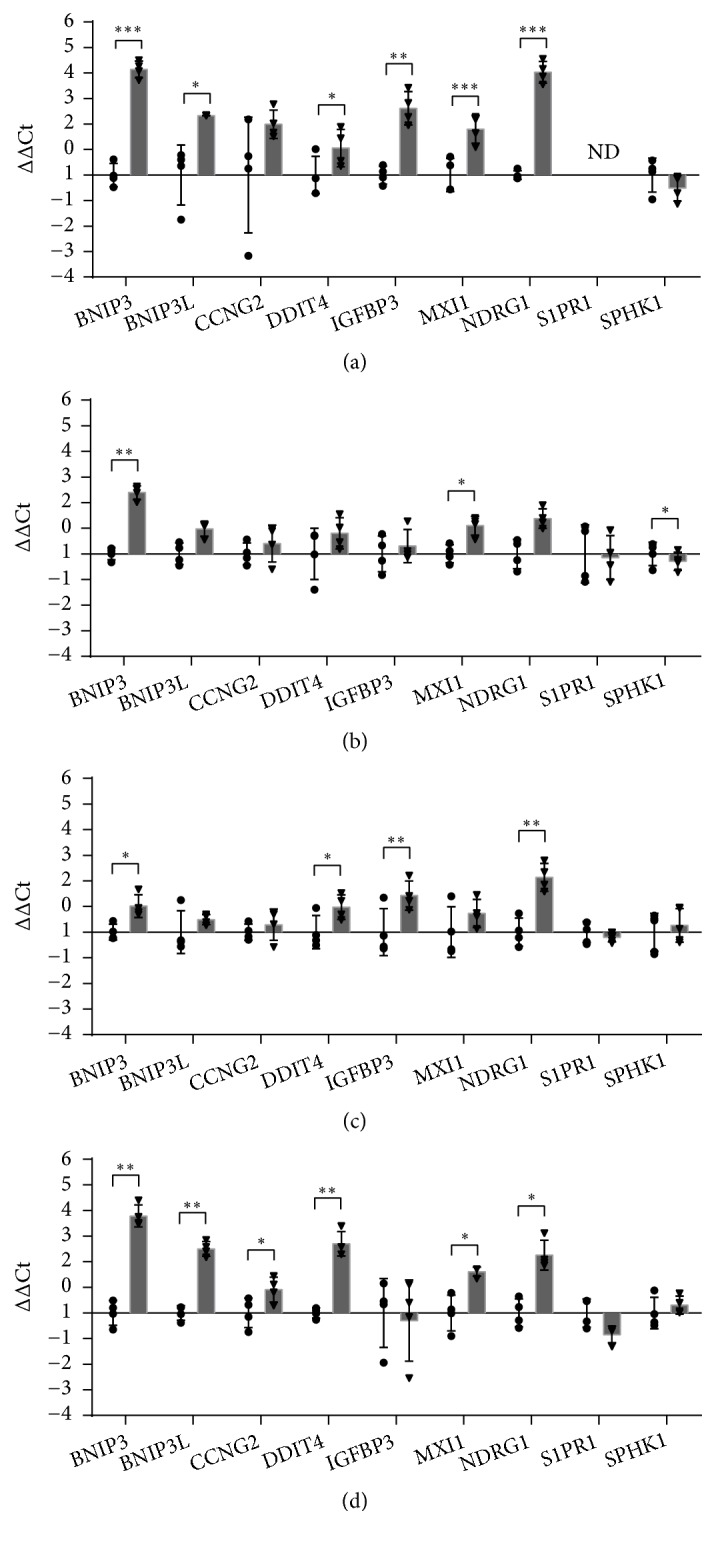
RT-qPCR analysis of genes involved in apoptosis and cell cycle after 24 hours of incubation in normoxia or hypoxia in HaCaT (a), HDF (b), HMEC-1 (c) and THP-1 (d). The results are expressed as ∆∆Ct after normalization on RPLP0 housekeeping gene. Data are shown as mean ± standard deviation and as single values distribution of four independent experiments. Circles (●) and triangles (▼) represent ∆∆Ct values in normoxia and hypoxia, respectively. Statistical analysis was performed using the two-tailed Student t-test comparing, for each gene, the expression in hypoxia versus normoxia (*∗*p-value < 0,05; *∗∗* p-value < 0,01; *∗∗∗*p-value < 0,001).

Usually, severe and prolonged hypoxia can induce apoptosis, whereas mild hypoxia (oxygen levels above 0.5%) prevents cells from undergoing apoptosis [43]. Under hypoxia, cells can arrest cell cycle at the G_1_/S interface [44] and several genes can be expressed to promote cell surviving. Moreover, hypoxia can reduce the sensitivity of cells to apoptotic stimuli [45]. Complex mechanisms stimulate the production of both pro- and antiapoptotic factors but also of factors that induce cell proliferation. Our data clearly show that hypoxia significantly affected the expression of genes involved in apoptosis and cell growth (Figure 4). In particular, hypoxia induced both proapoptotic and antiapoptotic-genes in all the tested cell lines, suggesting a fine balance between pro- and antiapoptotic signals, both responsible for cell-fate. The putative proapoptotic gene* BNIP3* and the closely related gene* BNIP3L* encode proteins which are members of the BH3-only subfamily of Bcl-2 [46] able to antagonise the activity of prosurvival proteins, such as Bcl-2 [47]. Hypoxia increased BNIP3 gene expression in all the tested cell lines and BNIP3L in HaCaT and THP-1 (Figure 4). BNIP3 and BNIP3L have been previously reported to promote cell death [48]. However, the concomitant expression of BNIP3 and BNIP3L seems critical for autophagy induction as part of a general mechanism of cell survival [49]. Therefore, the expression of these genes can contribute to cell survival, through the induction of autophagy, rather than of cell death.* IGFBP3*(Insulin Like Growth Factor Binding Protein 3), which was significantly induced in HaCaT and HMEC-1 (Figures 4(a) and 4(c)), encodes another proapoptotic protein. Previous studies have already shown the* in vitro* induction of* IGFBP3* mRNA under hypoxia in different cell lines, including HMEC-1 [50].* MXI1* (MAX-interacting protein 1) which was significantly overexpressed in HaCaT, HDF, and THP-1 encodes an antagonist of C-Myc, a transcription factor regulating the expression of genes involved in cell growth and apoptosis. Increased MXI-1 expression leads to growth arrest [51] and energy metabolism reprogramming in cancer cells [52]. The enzyme encoded by* SPHK1*(sphingosine kinase 1) catalyses the phosphorylation of sphingosine to form sphingosine-1-phosphate. While ceramide and sphingosine are usually proapoptotic, sphingosine-1-phosphate stimulates growth and cell survival [53]. Our data show that* SPHK1* is downregulated in HaCaT and HDF cells (Figures 4(a) and 4(b)).

Stress responsive proteins are usually produced to enhance the survival of cells exposed to environmental stress, including hypoxia. The expression of the stress-response gene* DDIT4* (DNA damage Inducible Transcript 4) is induced by hypoxia by coactivation of HIF-1*α* and Sp1 [54]. Our data showed that it was up-regulated by hypoxia in HaCaT, HMEC-1 and differentiated THP-1 (Figures 4(a), 4(c) and 4(d)). DDIT4 can function as a pro- or antiapoptotic factor depending on the cellular context [55]. In HaCaT keratinocytes, DDIT4 exerts an anti-apoptotic role [56], since its downregulation is essential for apoptotic program induction, suggesting in turn that its upregulation may protect cells from apoptosis. Cyclin G2 is a conserved cyclin encoded by the CCNG2 gene, which is highly expressed in the immune system [57]. Cyclin G2 induces a p53-dependent cell cycle arrest [58] and it is strongly upregulated during G_1_ and G_2_-phase in response to cellular stresses and growth inhibitory signals [57]. Our data showed its induction only in THP-1 cell lines (Figure 4(d)).* NDRG1*(N-myc downstream regulated 1), overexpressed in HaCaT, HMEC-1 and THP-1 cell lines (Figures 4(a), 4(c) and 4(d)), encodes a stress responsive protein that participate in the regulation of cellular differentiation, proliferation, growth arrest, apoptosis, angiogenesis and hypoxia sensing [59, 60]. In response to different insults,* NDRG1* expression is induced by HIF-1*α* [61], HIF-2*α* [62], and EGR-1/SP1 [63].

Altogether, these results indicate that hypoxia induced the expression of genes that may contribute to growth arrest rather than to cell death, in accordance to literature data [43].

### 3.5. Chemokines and Cytokines

**Figure 5:**
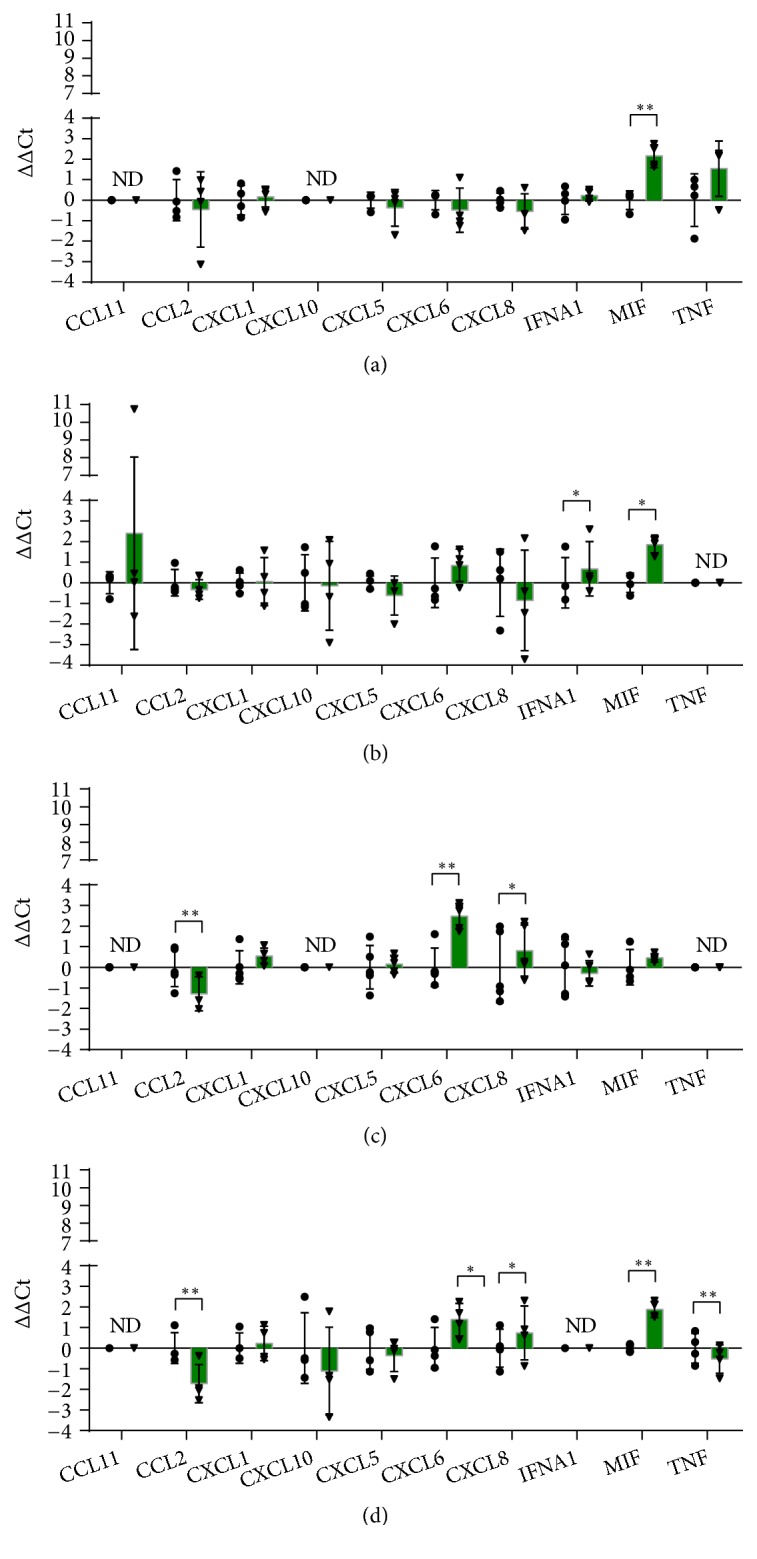
RT-qPCR analysis of genes encoding cytokines and chemokines after 24 hours of incubation in normoxia or hypoxia in HaCaT (a), HDF (b), HMEC-1 (c) and THP-1 (d). The results are expressed as ∆∆Ct after normalization on RPLP0 housekeeping gene. Data are shown as mean ± standard deviation and as single values distribution of four independent experiments. Circles (●) and triangles (▼) represent ∆∆Ct values in normoxia and hypoxia, respectively. Statistical analysis was performed using the two-tailed Student t-test comparing, for each gene, the expression in hypoxia versus normoxia (*∗*p-value < 0,05; *∗∗* p-value < 0,01).

Chemokines and cytokines play a key role in orchestrating the multistep process of wound healing through the regulation of angiogenesis and the recruitment of endothelial and inflammatory cells. Few genes encoding chemokines and cytokines were modulated by 24 hours of hypoxia (Figure 5). In HaCaT,* MIF* (Macrophage Migration Inhibitory Factor) was the only up-regulated gene. The expression of this gene was also increased in HDF and THP-1. MIF is a proinflammatory cytokine participating in the regulation of cell proliferation and differentiation. It is produced by a variety of cell types, including keratinocytes, monocytes, and endothelial cells [64, 65], and is induced by hypoxia [66], consistently with our results.* CXCL6* (C-X-C motif chemokine ligand 6) and* CXCL8* (C-X-C motif chemokine ligand 8) encode members of CXC chemokines. These chemotactic peptides are involved not only in leukocytes migration, but also in angiogenesis and inflammation. CXCL6 and CXCL8, being ERL+ chemokines, are potent angiogenic factors [67], able to directly induce endothelial cells migration and proliferation [68]. Here, the expression of CXCL6 and CXCL8 was increased by hypoxia in HMEC-1 and in THP-1 (Figures 5(c) and 5(d)). The increased CXCL8 gene expression in HMEC-1 is consistent with data from Karakurum** et al. [69]**but in contrast to the effect observed by Loboda and colleagues** [21]**Increased expression of CXCL8 by mouse and human macrophages has been already described** [70]**.

*CCL2* (C-C motif chemokine ligand 2) gene encodes a member of the CC chemokine family, also known as Monocyte Chemoattractant Protein 1, able to attract macrophages. CCL2 gene expression was down regulated by hypoxia in HMEC-1 and THP-1 (Figures 5(c) and 5(d)). Downregulation of CCL-2 expression by hypoxia has been previously demonstrated in other cell types [71, 72]. This effect may suggest a beneficial role, since a prolonged inflammatory response, mediated by macrophages, can lead to a chronic nonhealing wound.

TNF-*α* is a proinflammatory cytokine involved in the early phases of wound healing. Macrophages may polarize along proinflammatory macrophages (M1) and anti-inflammatory macrophages (M2) [73]. In our model,* TNF* gene expression was significantly downregulated in THP-1 by hypoxia (Figure 5(d)). This may suggest that hypoxia contribute to the differentiation of macrophages into an M2 subtype (M2d) characterized by an angiogenic phenotype [74]. M2d macrophages express high levels of IL-10 and VEGF and low levels of TNF-*α*. It seems thus that hypoxia, through the down regulation of CCL2 and TNF-*α*, contribute to the establishment of an anti-inflammatory environment needed for promoting wound healing.

However, the upregulation of IFNalpha by hypoxia in HDF may suggest a detrimental role of hypoxia in wound healing, since IFN-alpha injection reduced healing in a mouse model [75].

### 3.6. Growth Factors and Receptors

**Figure 6:**
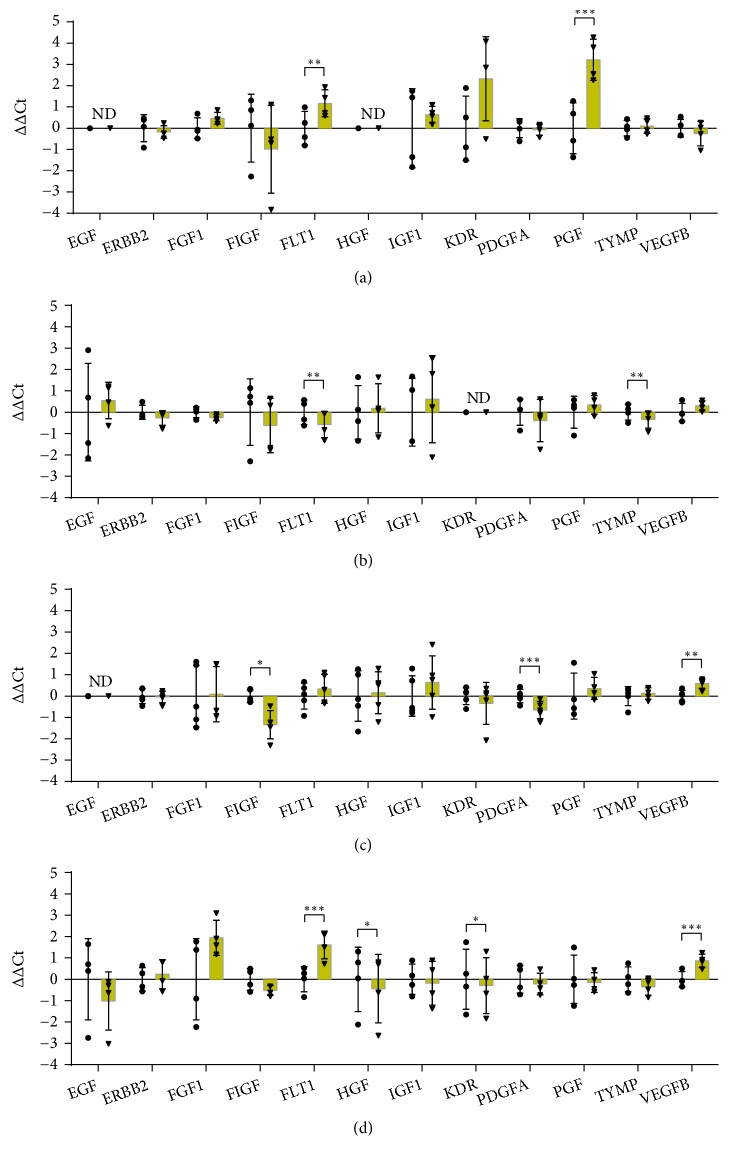
RT-qPCR analysis of genes encoding growth factors/growth factor receptors after 24 hours of incubation in normoxia or hypoxia in HaCaT (a), HDF (b), HMEC-1 (c), and THP-1 (d). The results are expressed as ∆∆Ct after normalization on RPLP0 housekeeping gene. Data are shown as mean ± standard deviation and as single values distribution of four independent experiments. Circles (●) and triangles (▼) represent ∆∆Ct values in normoxia and hypoxia, respectively. Statistical analysis was performed using the two-tailed Student's t-test comparing, for each gene, the expression in hypoxia versus normoxia (*∗*p-value < 0,05; *∗∗* p-value < 0,01; *∗∗∗*p-value < 0,001).

In addition to VEGFA, several genes coding growth factors and receptors were analysed (Figure 6). Modulation of the expression of these genes by hypoxia was cell type-specific. Some growth factors and receptors were up-regulated whereas others were downregulated by hypoxia.

*FLT1* and* KDR* encode VEGF receptor 1 and VEGF receptor 2, respectively. VEGFA binds both receptors, even if all the VEGFA effects seem predominantly mediated by KDR [76]. Moreover, FLT-1 possesses higher affinity than KDR for VEGFA, thus acting as a decoy receptor and sequestering VEGFA [77]. PGF (placental growth factor, a member of the VEGF family) and VEGF-B bind FTL-1, but not KDR. Interestingly,* FLT1* gene expression was increased, while* KDR* was decreased by hypoxia in differentiated THP-1 (Figure 6(d)).* VEGFB* was also up-regulated in THP-1 suggesting that VEGF-B could act on monocytes in an autocrine way.* FLT1* was also up-regulated in HaCaT, where its ligand (*PGF*) was up-regulated, too (Figure 6(a)). PGF is a potent angiogenic factor that is induced in keratinocytes during wound healing [78]. Here, we observed that gene expression of its receptor (*FTL1*) was up- regulated not only in HaCaT but also in THP-1 cells. PGF is a potent chemotactic factor for monocytes that could contribute to monocyte recruitment in the hypoxic microenvironment of wound healing.

The expression of different genes coding for angiogenic factors was downregulated by hypoxia.* HGF* (Hepatocyte growth factor) and* TYMP* (Endothelial cell growth factor 1) gene expression was decreased in THP-1 and in HDF, respectively (Figures 6(d) and 6(b)). The expression of* PDGFA* (Platelet Derived Growth Factor Subunit A) and* FIGF* (VEGFD) was down regulated by hypoxia in HMEC-1 (Figure 6(c)).

Angiogenesis in wound healing is a regulated process with a specific temporal production of different pro- and antiangiogenic factors. Our data suggest that hypoxia can differently induce or decrease the production of these factors in a cell-specific way.

### 3.7. Coagulation

**Figure 7:**
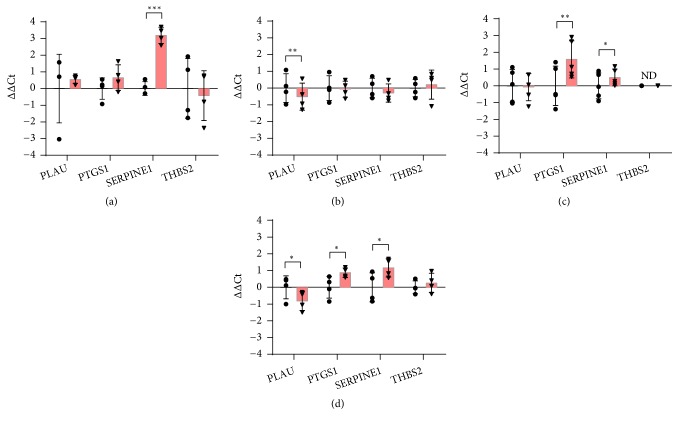
RT-qPCR analysis of genes involved in coagulation after 24 hours of incubation in normoxia or hypoxia in HaCaT (a), HDF (b), HMEC-1 (c), and THP-1 (d). The results are expressed as ∆∆Ct after normalization on RPLP0 housekeeping gene. Data are shown as mean ± standard deviation and as single values distribution of four independent experiments. Circles (●) and triangles (▼) represent ∆∆Ct values in normoxia and hypoxia, respectively. Statistical analysis was performed using the two-tailed Student t-test comparing, for each gene, the expression in hypoxia versus normoxia (*∗*p-value < 0,05; *∗∗* p-value < 0,01; *∗∗∗*p-value < 0,001).

Since both anti- and procoagulant factors are transcriptional targets of HIF-1 [79], some genes involved in coagulation were analysed.* PTGS1* (Prostaglandin-endoperoxide synthase 1), whose expression was increased by hypoxia in HMEC-1 and THP-1 (Figures 7(c) and 7(d)), encodes the constitutive isoform of cyclooxygenase that catalyses the conversion of the arachidonic acid into prostaglandins. In particular, this isoform generates prostaglandin E_2_ and prostaglandin D_2_, which are not only important for vascular homeostasis, but also for inducing the expression of some growth factors of the VEGFs family, promoting new vessels formation during skin repair [80, 81].

*PLAU* encodes urokinase plasminogen activator (uPA), which cleaves plasminogen into plasmin.* SERPINE1* (Serpin family E member 1) encodes Plasminogen activator inhibitor-1 (PAI-1), a serine proteinase inhibitor of uPA and tissue plasminogen activator (tPA). Both uPA and PAI-1 play a key role in coagulation and tissue remodeling. PAI-1 and uPA are usually secreted by several cell types including endothelial cells, macrophages and fibroblasts. Previous studies have shown that hypoxia up-regulates PAI-1, whereas it downregulates uPA [82, 83]. Here,* SERPINE1* gene expression was significantly up-regulated by hypoxia in HaCaT, HMEC-1 and differentiated THP-1 (Figures 7(a), 7(c), and 7(d)) whereas* PLAU* was downregulated in HDF and THP-1 (Figures 7(b) and 7(d)). The fine balance between PAI-1 and uPA is required for efficient tissue repair. In chronic wounds the excessive secretion of PAI-1 leads to the inhibition of uPA with the subsequent formation of fibrotic tissue [84].

### 3.8. Transcription and Signaling

**Figure 8:**
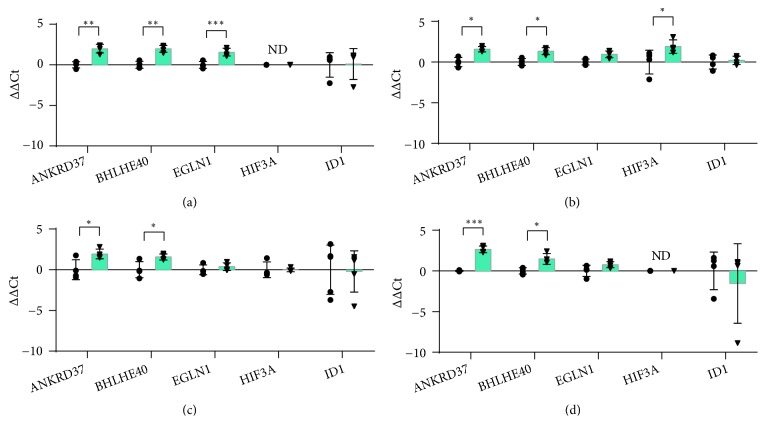
RT-qPCR analysis of genes involved in transcription and signaling after 24 hours of incubation in normoxia or hypoxia in HaCaT (a), HDF (b), HMEC-1 (c) and THP-1 (d). The results are expressed as ∆∆Ct after normalization on RPLP0 housekeeping gene. Data are shown as mean ± standard deviation and as single values distribution of four independent experiments. Circles (●) and triangles (▼) represent ∆∆Ct values in normoxia and hypoxia, respectively. Statistical analysis was performed using the two-tailed Student's t-test comparing, for each gene, the expression in hypoxia versus normoxia (*∗*p-value < 0,05; *∗∗* p-value < 0,01; *∗∗∗*p-value < 0,001).

Since the adaptive response to hypoxia allows for the transcriptional activation of multiple genes, five genes encoding transcription factors or molecules which interact with transcription factors were included in the analysis (Figure 8).

ANKRD37 (Ankyrin repeat domain 37) and BHLHE40 (Basic helix-loop-helix family member e40) were up-regulated in all the cell types analysed.* ANKRD37* encodes a short protein with unknown function but characterised by ankyrin repeats, which are known to be involved in the regulation of the activity of transcription factors such as NF-*κ*B and p53 [85].* ANKRD37*is known as a target of HIF-1 in different cell lines [86]. Since it was significantly induced in all cell lines tested in this work (Figure 8), it is likely to have an important role in the transcriptional program induced by hypoxia in the skin. This may be true also for* BHLHE40*(DEC1), which encodes a transcriptional repressor involved in adaptation to hypoxia [87]. Differently from* ANKRD37*, the induction of* BHLHE40* in response to hypoxia occurs through a p53-dependent mechanism, independent from HIF1-*α*. Since* BHLHE40* may contribute to muscle regeneration after ischemia [88], a role also in skin wound healing is plausible.

Two genes are strictly connected with HIF activity:* EGLN1* and* HIF3A*.* EGLN1* encodes PHD2, one of the three isoforms of human prolyl hydroxylases. Prolyl hydroxylation is a critical event to initiate oxygen-dependent degradation of HIF1-*α* in normoxia. PHD2 regulates the homeostatic levels of HIF-1*α* and is a cellular oxygen sensor [89, 90].* EGLN1* was significantly up-regulated only in HaCaT (Figure 8(a)).* HIF3A* encodes HIF-3*α*, a transcription factor which is related to HIF-1*α* both structurally and for common responsive elements [91]. In contrast to the ubiquitarian HIF-1*α*, HIF-3*α* appears to be expressed in a cell type-specific manner. Our data show that* HIF3A*was not expressed in HaCaT and differentiated THP-1, and it was significantly upregulated in HDF cells only (Figure 8(b)), in accordance with previous works [92].

*ID1 *(inhibitor of DNA-binding type 1) encodes a member of helix-loop-helix (HLH) proteins and inhibits the transcription activity of other HLH proteins [93].* ID1*, which favours cell migration and proliferation, is up-regulated upon skin injury and downregulated during wound repair [94, 95]. However, in the tested cell types, including HaCaT keratinocytes,* ID1* was not significantly modulated by hypoxia. This supports that* ID1* modulation requires interaction between cells, as previously described [94, 95].

### 3.9. Glycolytic Metabolism

**Figure 9:**
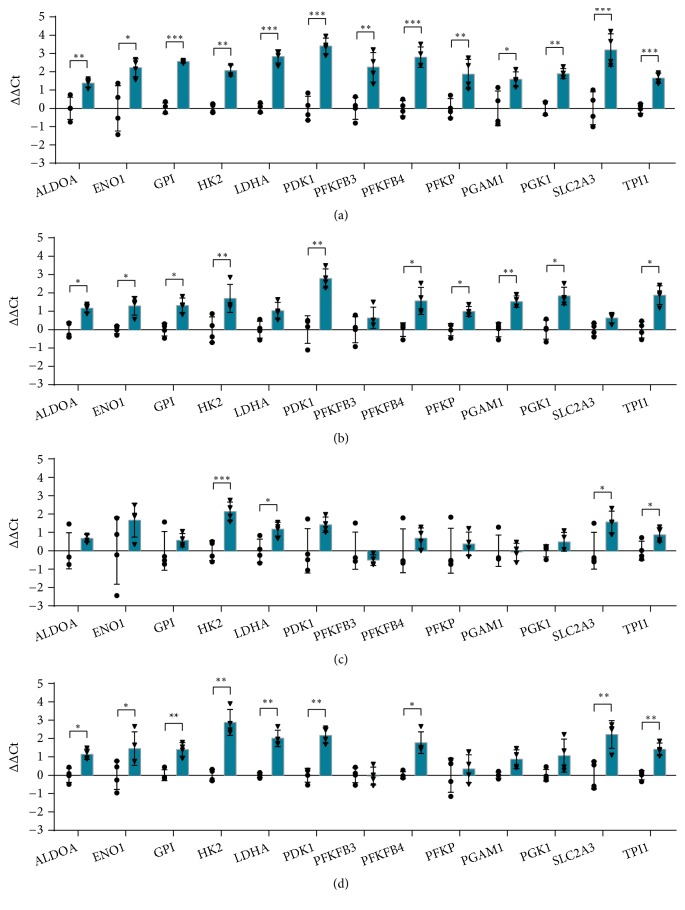
RT-qPCR analysis of genes involved in glycolytic metabolism after 24 hours of incubation in normoxia or hypoxia in HaCaT (a), HDF (b), HMEC-1 (c) and THP-1 (d). The results are expressed as ∆∆Ct after normalization on RPLP0 housekeeping gene. Data are shown as mean ± standard deviation and as single values distribution of four independent experiments. Circles (●) and triangles (▼) represent ∆∆Ct values in normoxia and hypoxia, respectively. Statistical analysis was performed using the two-tailed Student's t-test comparing, for each gene, the expression in hypoxia versus normoxia (*∗*p-value < 0,05; *∗∗* p-value < 0,01; *∗∗∗*p-value < 0,001).

Under hypoxia glycolysis is enhanced and may function as a compensatory mechanism for ensuring enough generation of ATP. Moreover, enhanced glycolysis leads to the increase of the end-product lactic acid, which promotes angiogenesis, enhances collagen deposition and accelerates wound healing [96, 97]. The genes encoding the enzymes involved in glycolysis are indeed upregulated, through HIF-1*α* stabilization [98]. The hypoxia-responsive genes controlling the shift from mitochondrial oxidative phosphorylation to glycolytic metabolism are expected to be shared by different cell populations. Yet, our data show some differences in gene expression in the different cell types. All the 13 genes considered in this study were significantly increased in HaCaT keratinocytes (Figure 9(a)). Ten and 9 genes were upregulated in HDF and THP-1 respectively (Figures 9(b) and 9(d)), while the expression of 4 genes was increased in HMEC-1 (Figure 9(c)). The gene encoding hexokinases 2 (*HK2*), an important enzyme responsible for the catalysis of the first step of the glycolytic pathway, that is the phosphorylation of glucose into glucose-6-phosphate, was significantly increased by hypoxia in all the tested cell lines (Figure 9). This result was expected, since HK2 is encoded by a HIF-1 target gene, unlike other HK isoforms [99].* GPI* (Glucose-6-phosphate isomerase) encodes the glycolytic enzyme that interconverts glucose-6-phosphate and fructose-6-phosphate. Extracellularly, the encoded protein functions as a lymphokine and angiogenic factor [100].* GPI* expression was significantly increased in all the cell types except HMEC-1 (Figure 9).* PFKP* (Phosphofructokinase), which encodes the enzyme that converts fructose 6-phosphate (Fru-6-P) into fructose 1,6-bisphosphate was significantly increased in HaCaT and HDF (Figures 9(a) and 9(b)). PFKP activity is regulated by the energetic status of the cell through the inhibitory effect of ATP, that limits glycolysis under aerobic conditions, and by the allosteric activation by fructose-2,6-bisphosphate (Fru-2,6-P_2_) [101, 102]. The synthesis of Fru-2,6-P_2_ from Fru-6-P is catalyzed by the proteins encoded by* PFKFB3 *and* PFKFB4* genes, which are induced by hypoxia via HIF-1*α* activation, as demonstrated by the discovery of HIF-1-binding sites within their promoters [103, 104]. These enzymes are known as 6-phosphofructo-2-kinase/fructose-2,6-bisphosphatase, which catalyse not only the synthesis but also the degradation of the glycolytic by-product Fru-2,6-P_2_. PFKFB3 shows the highest kinase/phosphatase activity ratio [105], thus enhancing glycolysis. Our data showed that* PFKFB3*was significantly up-regulated only in HaCaT cells (Figure 9(a)), opposite to* PFKFB4* which was induced in all the cell lines but HMEC-1. The protein encoded by* PGK1* (phosphoglycerate kinase 1) is a glycolytic enzyme that catalyses the conversion of 1,3-diphosphoglycerate into 3-phosphoglycerate, coupled with the synthesis of ATP from ADP.* PGK1* is a HIF1*α* target gene that can phosphorylate pyruvate dehydrogenase kinase 1 (*PDK1*), leading to inhibition of mitochondrial metabolism and improvement of glycolysis. During hypoxia, PGK1 is also involved in regulation of autophagy [106]. Here,* PGK1* gene expression was induced in HaCaT and HDF (Figures 9(a) and 9(b)), while* PDK1* was upregulated in HaCaT, HDF and THP-1 (Figures 9(a), 9(b) and 9(d)). PDK1 plays an important role also in proliferation, since it protects cells against apoptosis in response to hypoxia and oxidative stress, weakening the activity of respiratory chain [107].* LDH* (Lactate dehydrogenase) is a tetrameric enzyme composed by four subunits, the two most common of which are LDH-H, encoded by the* LDHB* gene, and LDH-M, encoded by the HIF-1 target gene* LDHA *and therefore induced under hypoxia. Compared to LDH-H, LDH-M preferentially catalyses the reduction of pyruvate into lactate [108], showing a pivotal role in sustaining high glycolytic flux and counteracting apoptosis. The increase of* LDHA *expression occurs in tandem with the inhibition of pyruvate dehydrogenase mediated by PDK1, diverting pyruvate from the tricarboxylic acid cycle. The conversion of pyruvate into lactate couples at the same time the oxidation of NADH to NAD^+^, restoring the pool required for glycolytic auto-sufficiency when oxygen becomes a limiting factor. Moreover, the resulting low levels of pyruvate enable cells relying on glycolysis to evade cell death [109]. LDHA was significantly up-regulated in HaCaT, HMEC-1 and HDF (Figures 9(a), 9(b), and 9(c)).

*SLC2A3*(Solute Carrier Family 2 Member 3), which was significantly induced in HaCaT, HMEC-1 and THP-1 cells (Figures 9(a), 9(b), and 9(c)), encodes Glucose transporter 3 (GLUT3), responsible for facilitating the diffusion of monosaccharides, in particular glucose, across the plasma membrane. The HIF-1-dependent expression of GLUT3 [110] plays an important role in ensuring efficient glucose uptake, even when glucose becomes a limiting factor [111], thus accomplishing the glycolytic switch seen under hypoxic conditions.

### 3.10. Nonglycolytic Metabolism

**Figure 10:**
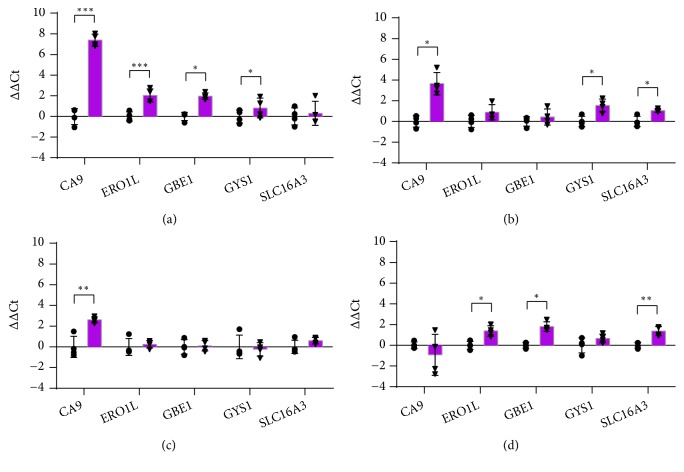
RT-qPCR analysis of genes involved in nonglycolytic metabolism after 24 hours of incubation in normoxia or hypoxia in HaCaT (a), HDF (b), HMEC-1 (c), and THP-1 (d). The results are expressed as ∆∆Ct after normalization on RPLP0 housekeeping gene. Data are shown as mean ± standard deviation and as single values distribution of four independent experiments. Circles (●) and triangles (▼) represent ∆∆Ct values in normoxia and hypoxia, respectively. Statistical analysis was performed using the two-tailed Student t-test comparing, for each gene, the expression in hypoxia versus normoxia (*∗*p-value < 0,05; *∗∗* p-value < 0,01; *∗∗∗*p-value < 0,001).

*CA9* encodes carbonic anhydrase 9, a transmembrane member of the zinc-metalloenzyme family that catalyses the reversible hydration of CO_2_, thus being involved in the regulation of pH homeostasis [112]. Due to the* Hypoxia Response Elements* (HREs) identified in its promoter, it is one of the most sensitive endogenous sensors of HIF-1*α* activity [113] and it has been proposed as an endogenous biomarker of cellular hypoxia in HMEC-1 [114]. Our data showed its significant induction in HaCaT, HDF and HMEC-1 (Figure 10).* ERO1L* (Endoplasmic reticulum oxidoreductase 1 alpha) encodes an endoplasmic reticulum membrane-associated oxidoreductase involved in disulphide bond formation [115], crucial for the proper folding of proteins.* ERO1L *appears to be upregulated by hypoxia and involved in VEGF secretion [116].* ERO1L* expression was significantly increased by hypoxia in HaCaT and THP-1 (Figures 10(a) and 10(d)).

Glycogen accumulation under hypoxic conditions seems to be an emerging metabolic survival pathway. Many cell lines contain a detectable amount of glycogen under normoxic culture conditions [117] and hypoxia induces accumulation of glycogen. Glycogen synthase 1 (*GYS1*) is responsible for the addition of glucose monomers to the growing glycogen molecule, whereas 1,4-*α* glucan branching enzyme 1 (*GBE1*) is responsible for the addition of branches. Their up-regulation during hypoxia is mediated by HIF-1 and induce glycogen accumulation [118]. In our cell lines,* GBE1* was up-regulated in HaCaT and in differentiated THP-1 while* GYS1* was up-regulated in HaCaT and HDF (Figure 10).

MCT4 protein, encoded by* SLC16A3 *(Solute carrier family 16 member 3), is a member of the monocarboxylate transporter family, which catalyses the bidirectional transport of short-chain monocarboxylates, such as L-lactate, pyruvate and ketone bodies, across the cell membrane. MCT4 is significantly expressed in hypoxic tissues, which depend on glycolysis for ATP production, and mediates the efflux of lactic acid from cells [119]. The expression of* SLC16A3* is up-regulated in response to hypoxia, through HIF-1-enhanced gene transcription [119]. In our model, SLC16A3 was significantly overexpressed by hypoxia in HDF and differentiated THP-1 (Figures 10(b) and 10(d)).

## 4. Conclusions

In this work, the changes in gene expression in response to hypoxic condition in cell populations involved in wound healing have been described. Under hypoxia, cells undergo a variety of biological modifications which can be different depending on the cell types, their function and energy requirements.

**Figure 11:**
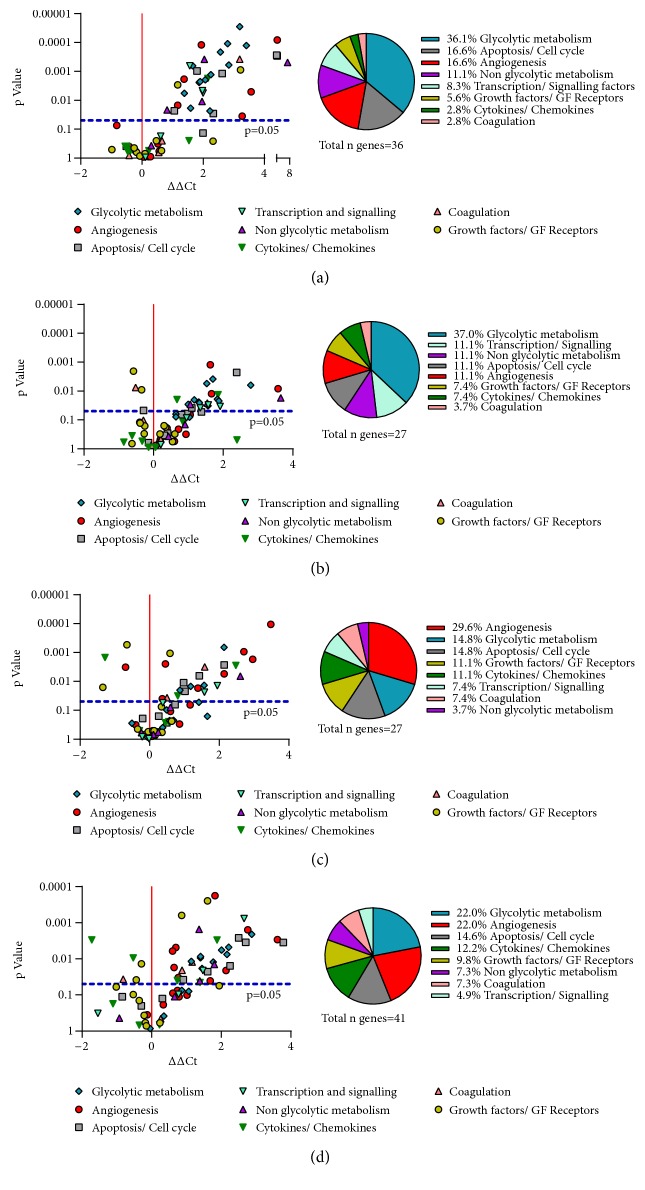
Volcano plot of the gene expression profiles and pie chart. The gene expression profiles was evaluated after 24 hours of incubation in normoxia or hypoxia in HaCaT (a), HDF (b), HMEC-1 (c) and THP-1 (d). In Volcano Plots each point represents the difference in expression between normoxia and hypoxia against the level of significance. The x-axis specifies the ΔΔCt and the y-axis specifies the negative logarithm to the base 10 of the t-test p-values. The horizontal dotted line reflects the filtering criteria (p-value = 0.05). Pie charts indicate the numerical proportion of each set of genes whose expression was modulated in hypoxia.

Exposure of different cell types to hypoxia revealed distinctive results, showing a higher number of genes modulated in HaCaT and differentiated THP-1 and a lower number of genes modulated in HDF and HMEC-1 (Figure 11). In HaCat and HDF, most of the modulated genes belong to the class of glycolytic metabolism. In these cell types, hypoxia mostly induce the expression of genes needed for reprogramming cells from oxidative to glycolytic metabolism. Differently, in HMEC-1 the highest number of genes modulated by hypoxia encode proteins involved in angiogenesis. It seems that during hypoxia autocrine signals are required for sustaining angiogenesis by endothelial cells. A high number of genes encoding proteins involved in angiogenesis were also up-regulated in differentiated THP-1. This is not unexpected, since macrophages are known to play a crucial role in the modulation of angiogenesis by the production of secreted molecules. Genes coding for cytokines/chemokines and growth factor were also mostly modulated in differentiated THP-1 and in HMEC-1. However, VEGF-A gene expression was increased in all the cells type involved in wound healing, indicating that this essential growth factor is induced by hypoxia in aspecific way.

Hypoxia has a key role in wound healing. Knowing which genes are modulated by hypoxia in cell populations involved in skin repair may be useful to start deeper investigations on the underlying mechanisms.

## Data Availability

The data used to support the findings of this study are included within the supplementary information file. Moreover, the data are available from the corresponding author upon request.

## Supplementary Materials

Supplementary Materials Raw data (ΔΔCt) from RT-qPCR experiments are presented in Supplementary Table 1.
